# Dissecting myosin-5B mechanosensitivity and calcium regulation at the single molecule level

**DOI:** 10.1038/s41467-018-05251-z

**Published:** 2018-07-20

**Authors:** Lucia Gardini, Sarah M. Heissler, Claudia Arbore, Yi Yang, James R. Sellers, Francesco S. Pavone, Marco Capitanio

**Affiliations:** 1 LENS—European Laboratory for Non-linear Spectroscopy, University of Florence, Via Nello Carrara 1, 50019 Sesto Fiorentino, Italy; 2 National Institute of Optics—National Research Council, Largo Fermi 6, 50125 Florence, Italy; 3 Laboratory of Molecular Physiology, National Heart, Lung, and Blood Institute, National Institutes of Health, Bethesda, MD 20892-8015 USA; 4 Department of Physics and Astronomy, University of Florence, Via Sansone 1, 50019 Sesto Fiorentino, Italy; 5 Hunan Provincial Key Laboratory of Protein Engineering in Animal Vaccines, Hunan Agricultural University, Changsha, Hunan 410128 China

## Abstract

Myosin-5B is one of three members of the myosin-5 family of actin-based molecular motors. Despite its fundamental role in recycling endosome trafficking and in collective actin network dynamics, the molecular mechanisms underlying its motility are inherently unknown. Here we combine single-molecule imaging and high-speed laser tweezers to dissect the mechanoenzymatic properties of myosin-5B. We show that a single myosin-5B moves processively in 36-nm steps, stalls at ~2 pN resistive forces, and reverses its directionality at forces >2 pN. Interestingly, myosin-5B mechanosensitivity differs from that of myosin-5A, while it is strikingly similar to kinesin-1. In particular, myosin-5B run length is markedly and asymmetrically sensitive to force, a property that might be central to motor ensemble coordination. Furthermore, we show that Ca^2+^ does not affect the enzymatic activity of the motor unit, but abolishes myosin-5B processivity through calmodulin dissociation, providing important insights into the regulation of postsynaptic cargoes trafficking in neuronal cells.

## Abstract (web summary)

Myosin-5B is an actin-based motor important for endosome recycling, but the molecular mechanism underlying its motility remains unknown. Here authors use single molecule imaging and high-speed laser tweezers to dissect the mechanoenzymatic properties of myosin-5B, which shows processive motility with peculiar mechanosensitivity.

## Introduction

Cells continuously experience and respond to mechanical stimuli^1^. Mechanosensitive proteins including members of the myosin superfamily respond to the spatial distribution, direction, strength, and duration of forces with changed mechanoenzymatic properties^1–7^. The mechanosensitivity of myosins with cellular transport^2,7^ is directly implicated in the ability to efficiently maneuver a diverse range of cargos in the dense actin cytoskeleton^8,9^. Aberrant transport function is linked to debilitating diseases in humans^10–12^, underlining the importance of myosin in cellular homeostasis.

Class-5 myosins are prototypic double-headed molecular motors present in almost every mammalian cell that transport their cargo to the plus end of actin filaments^13–18^. In the past years, sophisticated biophysical techniques and structural studies have been employed to understand the molecular details and the mechanosensitivity of the myosin-5-based transport^19–23^. The structure of the three myosin-5 paralogues (5A, 5B, and 5C) is predicted to be substantially similar^17,24,25^. They are composed by a motor domain (head) that binds actin and hydrolyzes ATP, a neck domain containing six calmodulin-binding IQ motifs, two coiled-coil dimerization domains and a cargo-binding globular tail. Despite the overall structural similarity, large differences in the sequence of the three paralogues suggest that their mechanochemical properties might differ significantly^17^. Single-molecule studies uniformly indicate that myosin-5A is a high duty ratio motor (i.e., each head is strongly bound to actin for the majority of the actomyosin ATPase cycle) that coordinates its catalytic motor domains to move processively in 36 nm steps on actin, a prerequisite for efficient cargo transport^19,26–28^. Myosin-5C in contrast is a low duty ratio motor, not processive on individual actin filaments and therefore must work in ensembles to establish processivity in vitro^29–31^. Very little information is available on the biophysical properties of myosin-5B. Previous studies indicate that myosin-5B has a duty ratio that may support the processive movement^32,33^. However, single-molecule studies of myosin-5B processivity are lacking as is information about its regulation by external forces. Revealing the mechanical properties of myosin-5B is essential to elucidating the mechanisms of myosin-5B-driven cargo transport in a variety of crucial cellular processes such as postsynaptic plasticity^34^, epithelial cell polarization^35^, and unconventional long-range transport of vesicles organized in extensive actin networks^36^. Mutations in myosin-5B that disrupt its transport function lead to life-threatening pathologies such as microvillus inclusion disease, a rare human disease characterized by the lack of apical microvilli on intestinal epithelial cells^10,11,37^. Moreover, myosin-5B is expected to be regulated by calcium through the six calmodulins bound to its neck domain. In particular, during long-term potentiation of synaptic strength, myosin-5B transport of receptors and recycling endosomes is triggered by Ca^2+^ spikes^34^. Although Ca^2+^ has been shown to activate myosin-5B ATPase activity in vitro through the relief of an autoinhibited conformation^34^, no data are available about the effect of Ca^2+^ on myosin-5B motility so far.

Here, we implement advanced single-molecule tools to characterize the biophysical properties of myosin-5B and its regulation by force and calcium. We apply a fluorescence-based in vitro motility assay to investigate the processivity of myosin-5B motors under unloaded conditions. We demonstrate that myosin-5B moves processively in 36 nm steps on individual actin filaments as single motor. We then investigate load-dependence of myosin-5B movements with ultrafast force-clamp spectroscopy, a sub-millisecond and sub-nanometer resolution technique based on laser tweezers^38,39,40^. We find that myosin-5B step size, velocity, and run length are strongly mechanosensitive. Resistive forces progressively decrease myosin-5B velocity and run length up to stall at about 2 pN, where forward and backward stepping reach equilibrium. The motor directionality is reversed for forces >2 pN. On the other hand, assistive forces moderately affect myosin velocity, but strongly accelerate the detachment of the motor from the actin filament. We show that myosin-5B, although processive, is not as strong as myosin-5A as a single motor and probably evolved to efficiently transport cargoes in ensembles. Finally, we show that Ca^2+^ finely regulates myosin-5B motility in vitro by uncoupling the mechanical and enzymatic activity of the motor, giving insight into its transport function in neuronal cells. Our study provides mechanistic insight into the molecular basis underlying myosin-5B-based transport and offers a detailed model of its trafficking function in the crowded actin cytoskeleton of mammalian cells.

## Results

### Myosin-5B is a processive motor that moves in 36 nm steps

**Fig. 1:**
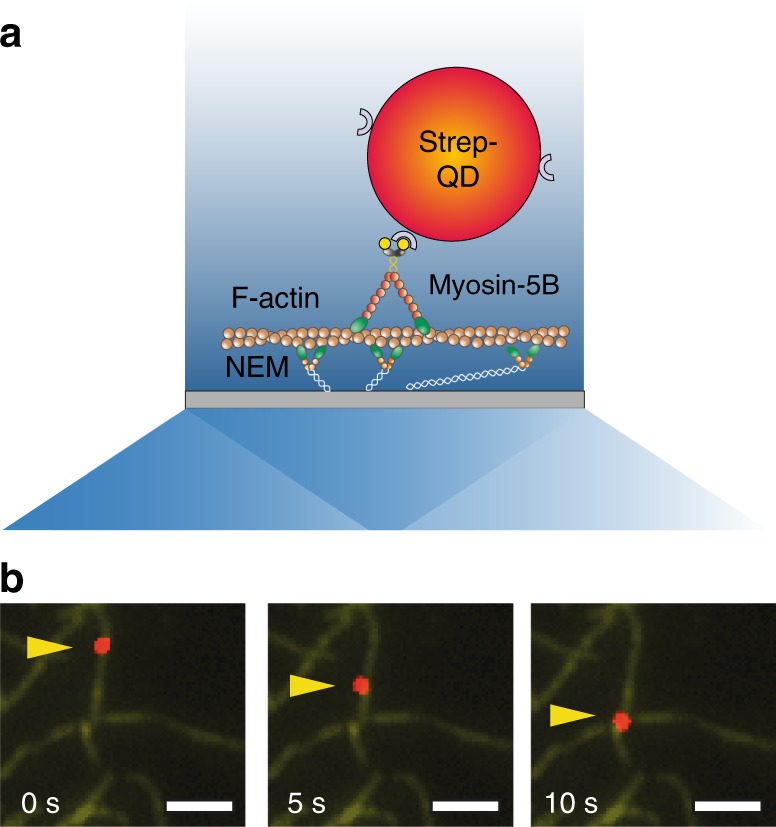
Processive movement of myosin-5B on F-actin in the single-molecule in vitro motility assay. **a** Schematic of the myosin-5B single molecule in vitro motility assay. A single biotinilated myosin-5B conjugated with a streptavidinated quantum dot (strep-QD) interacts with a single-actin filament. Actin filaments are anchored to the coverslip surface through *N*-ethylmaleimide modified myosin II (NEM). Schematic not drawn to scale. **b** Three consecutive frames (5 s interval) of a processive run of QD-labeled myosin-5B (red) on an actin filament (green). The yellow arrow indicates the QD position. Scale bar is 2 µm

A single-molecule in vitro motility assay was performed to establish whether myosin-5B is a processive motor on individual actin filaments under unloaded conditions. The movement of quantum dot (QD)-labeled myosin-5B heavy meromyosin (hereafter myosin-5B) on actin filaments was recorded on a custom-built multicolor total internal reflection fluorescence (TIRF) microscope (Fig. 1a, Methods section)^41^. This allowed us to simultaneously image actin and myosin-attached QDs in the evanescent field in close proximity to the coverslip with high spatiotemporal resolution (~4 nm in 100 ms, Fig. 1b).

**Fig. 2:**
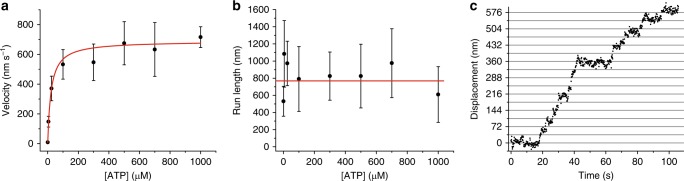
Signatures of single myosin-5B molecule motility under unloaded condition. **a** The velocity of myosin-5B movement (*n* = 127) on F-actin increases with increasing ATP following Michaelis–Menten kinetics (red curve), with *v*_max_ = 691 ± 25 nm s^−1^ and *K*_app_ = 22 ± 2 µM. Error bars, s.d. **b** The run length (*n* = 127) is ATP-independent within error bars. Average run length is 780 ± 2 nm (red line). Error bars, s.d. **c** Representative time trace of myosin-5B stepping forward in discrete 36 nm increments on the actin filament. Average step 36 ± 4 nm (*n* = 42, s.e.m., [ATP] = 0.3 µM)

The position of the QD was determined by fitting its point-spread-function with a 2D-Gaussian function using a custom-written Matlab script^41^. The coordinates of the center of the Gaussian function at each frame were plotted on a *x*–*y* plot from which motor trajectory, run length, and velocity were extracted (Methods). Figure 2a, b shows the dependence of myosin-5B velocity and run length on ATP concentrations from 0.3 to 1000 µM. The unloaded velocity follows Michaelis–Menten kinetics (Fig. 2a), with a maximum velocity *v*_max_ = 691 ± 25 nm s^−1^ and a *K*_app_ = 22 ± 2 µM, the ATP concentration at which the velocity is *v*_max_/2. The run length is with an average value of 780 ± 2 nm independent of the ATP concentration (Fig. 2b). Analysis of the motility data at 0.3 µM ATP revealed that a single myosin-5B moves processively in discrete 36 ± 4 nm steps when coupled to a QD in vitro (Fig. 2c).

We also measured the rate at which myosin-5B translocates actin in the in vitro gliding assay, in which multiple myosins attached to the coverslip surface moved fluorescently-labeled actin filaments^42^. An actin gliding velocity of 381 ± 45 nm s^−1^ (*n* = 379, [ATP] = 1000 µM) was obtained, demonstrating that multiple myosin-5B motors can work in large ensembles while maintaining fast processivity to translocate actin filaments, although slower than the single motor. Similar reduction in velocity has been previously observed for myosin-5A in the gliding assay compared to single-molecule values^43^ and in a two-motor motility assay^44^ and accounted for by the coupling between multiple myosins^45^.

### Myosin-5B velocity and run length dependence on load

To investigate how myosin-5B processivity is regulated by pN-range loads usually experienced inside cells, we adapted ultra-fast force-clamp spectroscopy^38^ to the study of processive molecular motors.

**Fig. 3:**
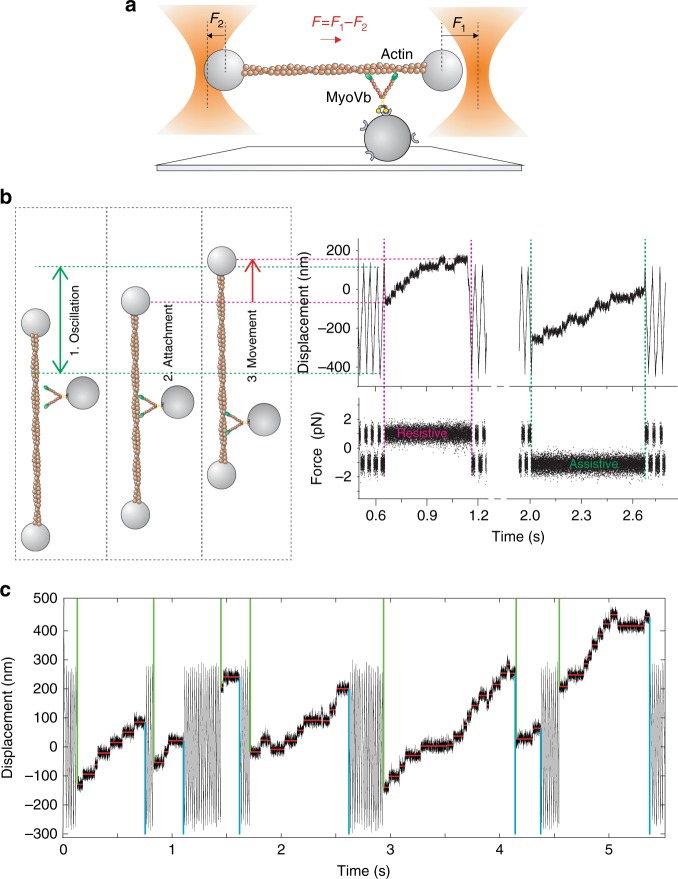
Load dependence of myosin-5B stepping signatures investigated with high-speed optical tweezers. **a** Schematic of ultrafast force-clamp spectroscopy applied to a processive myosin-5B motor. A single myosin-5B molecule is attached to a glass bead pedestal through a streptavidin-biotin link. A single actin filament is trapped by suspending it between alpha-actinin coated beads. Black arrows represent the force clamped on the right (*F*_1_) and left bead (*F*_2_), red arrow represents the net force (*F*) on the dumbbell. *F* is alternated back and forth to maintain the dumbbell within a limited oscillation range when myosin is not bound to actin. **b** Left: cartoon showing the position of the right bead when: (1) myosin is unbound and the dumbbell oscillates; (2) myosin attaches to actin; and (3) myosin moves towards the actin barbed end. Right: example trace showing displacement and force during the corresponding phases of dumbbell oscillation, myosin-5B attachment, and processive runs under assistive and resistive loads. **c** Position record showing myosin-5B processive runs and the step and run detection algorithm. Detected beginning and end of each run are indicated by green and cyan vertical lines, respectively. Red horizontal lines indicate the detected steps. [ATP] = 100 μM

For the assay, a single-actin filament was suspended between two optically trapped beads and brought in close proximity to a single myosin that was attached to a micron-sized glass bead on the coverslip surface (Fig. 3a, Methods section). A constant force was applied to the bead–actin–bead complex (herein named dumbbell) through a double feedback system. The dumbbell consequently moved at constant velocity against viscous drag from the solution. The force was alternated back and forth to oscillate the dumbbell in a triangular wave fashion within a user-defined spatial interval (Fig. 3b, 1. oscillation). When myosin attached to the actin filament, the force was rapidly transferred to the molecule and consequently, the dumbbell stopped its movement (Fig. 3b, 2. attachment).

We observed that a large proportion of actin–myosin interactions were composed of processive runs in which myosin-5B moved in discrete steps towards one end of the actin filament (Fig. 3b, 3. movement). The constant force exerted by the optical tweezers was assistive or resistive the protein movement depending on the force direction at the moment of attachment. The optical tweezers technique, thus allowed investigation of the dependence of processive myosin-5B runs under various intensities and directions of load. Ultra-fast force-clamp spectroscopy allows investigation of myosin movements under constant force rapidly after the actin–myosin bond is established^38^. This feature applied to processive motors results in quantification of myosin-5B run length and duration under different load regimes with high spatiotemporal accuracy. Moreover, the high rate of the force feedback (200 kHz) is crucial in mantaining constant force during rapid changes like the myosin working stroke (Fig. 3b). Lastly, the assay geometry, combined with nm-stabilization of the sample (Methods), assures fixed orientation of the force relative to the actin filament direction during myosin stepping.

**Fig. 4:**
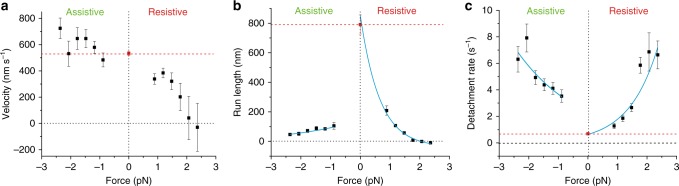
Myosin-5B velocity and run length and detachment rate from actin are force sensitive. Force dependence of myosin-5B average velocity (**a**), run length (**b**), and detachment rate (**c**). Detachment rate is calculated as *k* = 1/<*τ*>, where <*τ*> is the average run duration. In all panels, assistive forces are negative, resistive forces are positive. Red squares are the unloaded velocity (**a**), run length (**b**), and detachment rate (**c**) measured with the single-molecule motility assay (*n* = 30). Cyan curves in **b** and **c** are fits of the exponential model equations $L = L_0^ \pm {\mathrm{exp}}\left( { - \frac{{F \cdot d_L^ \pm }}{{{{k}}_{\mathrm{B}} \cdot {{T}}}}} \right)$ and $k = k_0^ \pm {\mathrm{exp}}\left( {\frac{{F \cdot d_k^ \pm }}{{{{k}}_{\mathrm{B}} \cdot {{T}}}}} \right)$to the data, where *F* is the absolute value of the force, *k*_B_ the Boltzmann constant, *T* the temperature, and + or − superscripts indicate free parameters under positive or negative force, respectively. Fitted parameters are *L*_0_^−^ = 148 ± 12 nm, *L*_0_^+^ = 890 ± 120 nm, *d*_*L*_^−^ = 1.9 ± 0.3 nm, *d*_*L*_^+^ = 6.3 ± 0.6 nm and *k*_0_^−^ = 2.3 ± 0.4 s^−1^, *k*_0_^+^ = 0.64 ± 0.09 s^−1^, *d*_*k*_^−^ = 1.9 ± 0.5 nm, *d*_*k*_^+^ = 4.2 ± 0.4 nm. Fisher *f*-test applied to run length and detachment rate data under positive and negative force gave *p* value = 2E−5 and 6E−3, respectively, indicating significant difference of myosin-5B mechanosensitivity with force direction. [ATP] = 100 µM. *n* = 756. Error bars (**a**, **b**), s.e.m. (**c**), s.e.m. obtained from error propagation *σ*_*k*_ *=* *σ*_*τ*_ *<* *τ* *>* ^−2^. Box plots of run length and detachment rate box plot are shown as Supplementary Fig. 1a, b

We separately analyzed myosin-5B runs under assistive and resistive forces from 0.5 to 3 pN and developed a step- and run-detection algorithm (Fig. 3c, Methods section) to determine run length and velocity. Figure 4 and Supplementary Fig. 1 show how force affects myosin-5B motility; data points at zero force (red squares and dotted lines) were obtained from the single-molecule motility assay (Fig. 1). Assistive forces slightly increase myosin velocity, whereas velocity and run length significantly decrease with resistive force. Both, velocity and run length vanish at the stall force of ~2 pN. Resistive forces >2–2.5 pN reverse the motor directionality. We observed a marked asymmetry of run length with respect to the direction of force (Fig. 4b). Myosin run length in the presence of 0.9 pN assistive force (105 ± 23 nm) was significantly shorter than for 0.9 pN force resisting the movement (209 ± 31), and even shorter than in the unloaded in vitro motility (780 ± 2 nm). From exponential fits to data ($L = L_0^ \pm {\mathrm{exp}}\left( { - \frac{{F \cdot d_L^ \pm }}{{k_{\rm{B}} \cdot T}}} \right)$) we obtained the distance parameters (*d*_*L*_^±^), which quantify the sensitivity of run length (*L*) to force (*F*), and the unloaded run lengths (*L*_0_^±^) under both assistive (^−^) and resistive (^+^) forces (*k*_B_ is the Boltzmann constant and *T* is the temperature). The unloaded run length for assistive forces was about sixfold shorter than for forces resisting the movement (*L*_0_^−^ = 148 ± 12 nm, *L*_0_^+^ = 890 ± 120 nm). On the other hand, the sensitivity of run length to force was markedly lower for assistive than for resistive forces (*d*_*L*_^−^ = 1.9 ± 0.3 nm, *d*_*L*_^+^ = 6.3 ± 0.6 nm). The separation between *L*_0_^−^ (148 ± 12 nm) and the run length measured with the unloaded motility assay (780 ± 2 nm) suggests that a highly force-sensitive transition occurs in the low assistive force range that we do not sample in our measurements (Supplementary Fig. 2).

To understand whether the observed run length mechanosensitivity was related to load-induced detachment of myosin-5B from actin, we plotted myosin detachment rates (i.e., the inverse of run durations) as a function of force (Fig. 4c). Detachment rates increase with force, supporting the idea that the reduction of run length with force is due to increased detachment from actin. Exponential fits of the detachment rate *k* vs force *F* ($k = k_0^ \pm {\mathrm{exp}}\left( {\frac{{F \cdot d_k^ \pm }}{{k_{\rm{B}} \cdot T}}} \right)$) further support this hypothesis and show that low assistive forces accelerate the detachment of myosin-5B from actin to a greater extent than resisting forces (*k*_0_^−^ = 2.3 ± 0.4 s^−1^, *k*_0_^+^ = 0.64 ± 0.09 s^−1^), and that the detachment of myosin-5B from actin is more sensitive to resistive than assistive forces (*d*_*k*_^−^ = 1.9 ± 0.5 nm, *d*_*k*_^+^ = 4.2 ± 0.4 nm). The distance parameters of run length (*d*_*L*_^−^) and detachment rate (*d*_*k*_^−^) under assistive force are identical, indicating that both quantities are governed by the detachment of myosin-5B from actin. On the other hand, *d*_*L*_^+^ > *d*_*k*_^+^, indicating that a process other than myosin detachment is at the base of the observed decrease in run length with resistive forces. In summary, our results indicate that myosin-5B motility is mechanosensitive and both run length and velocity are finely tuned by pN-forces in the cell. However, the data do not fully explain the molecular mechanisms underlying the mechanosensitivity of myosin-5B, particularly under resistive forces.

### Mechanosensitivity of myosin-5B stepping

**Fig. 5:**
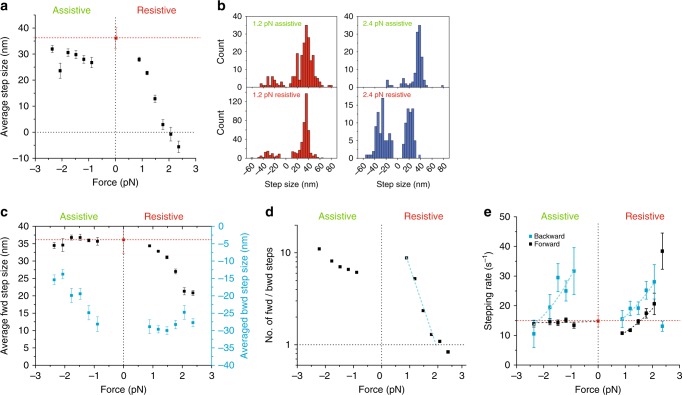
The ratio of forward to backward steps determines myosin-5B run length and velocity under resistive load. **a** Force dependence of the average step size. Box plot in Supplementary Fig. 1c. **b** Step size distributions at ±1.2 pN (left panel) and ±2.4 pN (right panel). For resistive forces close to the stall force (+2.4 pN), the distributions of forward (>0) and backward (<0) steps become nearly symmetric. **c** Force dependence of the average forward (black squares) and backward (cyan squares) step size. **d** Force dependence of the ratio of forward to backward steps. The dotted cyan line is the fit of the exponential model equation $R = R_0^ + {\mathrm{exp}}\left( { - \frac{{F \cdot d_{\rm{fwd/bwd}}^ + }}{{{{k}}_{\mathrm{B}} \cdot {{T}}}}} \right)$ to data for positive forces below the stall force, which gives *R*_0_^+^ = 54 ± 8 and *d*_fwd/bwd_^+^ = 8.2 ± 0.6 nm. **e** Force dependence of forward (black squares) and backward (cyan squares) stepping rates. Exponential fits $k_{f,b} = k_{0f,b}^ \pm {\mathrm{exp}}\left( {\frac{{{{F}} \cdot {{d}}_{f,b}^ \pm }}{{{{k}}_{\mathrm{B}} \cdot {{T}}}}} \right)$ of forward (_*f*_) and backward (_*b*_) stepping rates for positive (^+^) and negative (^−^) forces gave: $k_{0f}^ +$ = 6.5 ± 0.6 s^−1^, $d_f^ +$ = 2.2 ± 0.3 nm; $k_{0b}^ +$ = 10 ± 2 s^−1^, $d_b^ +$ = 2.0 ± 0.4 nm. $k_{0f}^ -$ = 14.6 ± 0.8 s^−1^, ${\mathrm{d}}_f^ -$ = 0.05 ± 0.15 nm; $k_{0b}^ -$ = 54.1 ± 17.5 s^−1^; $d_b^ -$ = 2.4 ± 0.9 nm. Error bars, s.e.m., *n*_fwd_ = 2729, *n*_bwd_ = 689. [ATP] = 100 μM. Red squares and dotted lines in **a**–**c** and **e** are, respectively, the unloaded step size and stepping rate measured with the single molecule motility assay at [ATP] = 100 μM (*n* = 42)

To relate the observed changes in velocity and run length (Fig. 4) to the myosin-5B mechanochemical cycle, we plotted the average step size against force (Fig. 5a). Note that the average step size is the sum of the forward (positive sign) and backward (negative sign) steps and does not have a physical meaning with regards to the actin filament lattice. The average step size is relatively constant under assistive forces, but slightly smaller than the value obtained at zero force using the single-molecule motility assay (red square and dotted line). The average step size rapidly decreases under resistive force and vanishes at a stall force of ~2 pN (Fig. 5a). This behavior raises the question whether (i) the amplitude of single myosin-5B steps decreases in proportion with the applied force and vanishes at the stall force or (ii) a different distribution of positive and negative step sizes accounts for the observed data.

The position records obtained at ~2 pN resistive force demonstrate that the step size of individual stepping events, contrary to the average step size, does not dissipate at the stall force (Supplementary Fig. 3). This observation demonstrates that myosin can take forward and backward steps. Analysis of the step size distribution at various assistive and resistive forces shows that resistive forces increase the probability of backward stepping, thus reducing or nullifying the average step size (Fig. 5b). We therefore separated forward and backward steps to quantify their average amplitude and force dependence (Fig. 5c, black and cyan squares, respectively). The average forward step size does not vary significantly under assistive forces and is with ∼36 nm identical to the parameter obtained in the unloaded motility assay (red dotted line). Resistive forces progressively reduce the average forward step size up to ∼20 nm at the stall force (2 pN). The force-dependence of the average backward step size is symmetric to the forward step size: the absolute value of the backward step is constant at resistive forces (∼30 nm) and decreases with assistive forces to ∼15 nm at ∼−2 pN force. The observed short forward step size at high resistive force (20 nm) can be explained if the trail head would bind to an actin monomer next to the lead head instead of binding to the next actin pseudo-repeat. This would produce short inchworm-like steps under load, as previously reported for myosin-6^46^ and myosin-5A^47^. A similar mechanism might occur for short backward steps (15 nm) at high assistive force.

To quantify whether the distribution of forward and backward steps can account for the observed force-dependence of myosin-5B velocity and run length, we plotted the ratio between the number of forward and backward steps *N*_fwd_/*N*_bwd_ (Fig. 5d). *N*_fwd_/*N*_bwd_ is greatly reduced by a resistive load and is approximately equal to 1 at the stall force (2 pN), explaining why both run length and velocity vanish at the same force. Data under resistive forces were fitted by an exponential function, which gives *N*_fwd_/*N*_bwd_ = 54 ± 8 in the absence of load and a distance parameter *d*_fwd/bwd_^+^ = 8.2 ± 0.6 nm, larger than the distance parameter of myosin detachment rate (*d*_*k*_^+^ = 4.2 ± 0.4 nm). In conclusion, our data indicate that the marked decrease of myosin-5B velocity and run length under resistive loads is governed by an increase in backstepping, whereas run length mechanosensitivity under assistive loads is driven by enhanced detachment of myosin from actin. The higher number of steps per run for resistive forces compared to assistive forces further supports this conclusion (Supplementary Fig. 4).

Additional insight into the stepping mechanism of myosin-5B comes from load-dependence of stepping rates (Fig. 5e). Myosin-5B forward stepping under assistive forces is load-insensitive and undistinguishable from the stepping rate under zero force, obtained from the single molecule motility assay. Small resistive forces (~0.7 pN) slow down myosin-5B forward stepping to ~70% of the unloaded rate. However, forces >0.7 pN accelerate myosin-5B forward stepping rate, which is nearly doubled at the stall force. This is opposite to Myosin-5A stepping rate, which has been shown to slow down under resistive force^48–51^. Myosin-5B velocity and run length decrease with resistive force despite the increased stepping velocity. This result further demonstrates that the observed velocity and run length decrease is mainly driven by the large increase in the ratio of backward to forward stepping (Fig. 5d). As expected, backward stepping accelerates with increasing resistive force and slows down with increasing assistive forces, although discontinuity in the resistive and assistive curves about zero force suggests that two different transitions are involved.

### The effect of Ca^2+^ on myosin-5B processivity

**Fig. 6:**
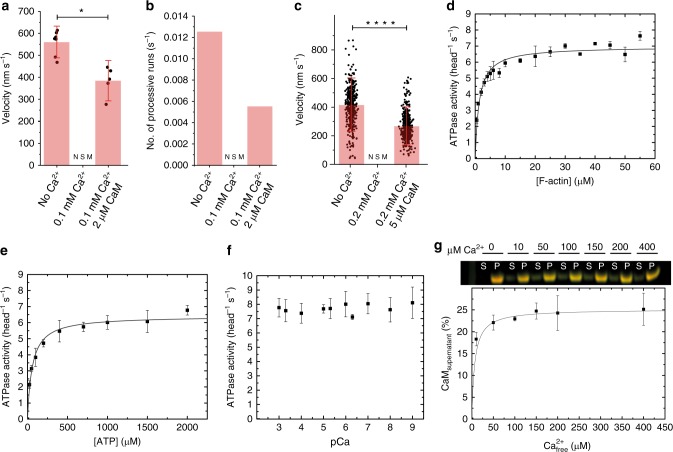
Effect of Ca^2+^ and CaM on single-molecule processivity and ensemble kinetics of myosin-5B. Dependence of myosin-5B velocity (**a**) and number of processive runs (**b**) on Ca^2+^ and CaM in the single-molecule motility assay. In this particular set of measurements, we observed 0.0125 runs s^−1^ with an average velocity of 559 ± 64 nm s^−1^ in the absence of Ca^2+^ and CaM. In the presence of 0.1 mM Ca^2+^, no significant movement (NSM) was observed. In the presence of 0.1 mM Ca^2+^ and 2 μM CaM, myosin processivity and velocity were partially rescued to 0.0055 runs s^−1^ and 383 ± 93 nm s^−1^ at [ATP] = 1000 μM (*n* = 12, *p* value = 0.02). Error bars, s.d. No error bars are reported in **b** because data are from single experiments. **c** Dependence of myosin-5B velocity on Ca^2+^ and CaM in the gliding assay. Myosin-5B translocated actin filaments with a speed of 413 ± 128 nm s^−1^ (*n* = 417) in the in vitro gliding assay. Filaments movement stalled (NSM) in the presence of 0.2 mM Ca^2+^ but was partially restored (264 ± 92 nm s^−1^, *n* = 439, *p* value = 4E−17) in the presence of 5 µM CaM. Error bars, s.d. Student’s *t* test applied on **a** and **c**, *P *<* 0.1, ****P *<* 0.0001. **d** Actin-activated steady-state ATPase activity of myosin-5B yielding the kinetic parameters *k*_cat_ = 6.96 ± 0.14 s^−1^ and *K*_app_ = 1.34 ± 0.16 µM (*n* = 3). **e** ATP-dependence of the steady-state ATPase activity of myosin-5B at 30 µM [F-actin] (*k*_cat,ATP_ = 6.41 ± 0.14 s^−1^; *K*_app,ATP_ = 59.07 ± 6.95 µM) (*n* = 3). **f** Increasing [Ca^2+^] does not affect the steady state ATPase of myosin-5B at a constant F-actin concentration of 30 µM (*n* = 3). **g** Quantification of CaM dissociation. Top, F-actin cosedimentation assays show that increasing concentrations of Ca^2+^ partially dissociate CaM from myosin-5B. The abbreviations S and P refer to supernatant and pellet fraction (full-length gel image in Supplementary Fig. 6). Bottom, densitometric analysis shows that on average 25% of the six CaM molecules dissociate with a *K*_D_ < 10 µM Ca^2+^ per myosin-5B heavy chain (*n* = 3)

Myosin-5B activity is dynamically activated by micromolar concentrations of Ca^2+^ in vivo and in vitro by the relief of autoinhibition^34^. To test if the mechanical activity of myosin-5B is Ca^2+^ sensitive, we performed single-molecule motility assays, gliding assays and optical trapping experiments in the presence of 100–200 μM Ca^2+^. The single molecule motility assay demonstrates that (i) processive runs disappear in the presence of free Ca^2+^ but (ii) that motility can be partially restored by excess CaM (Fig. 6a, b). In fact, the number of processive runs that we observed in a 1000 s time frame were 12.5 in the absence of Ca^2+^, zero in the presence of 100 μM Ca^2+^, and 5.5 in the presence of 100 μM Ca^2+^ and 2 μM CaM. Myosin-5B velocity followed a similar trend with an average value of 559 ± 64 nm s^−1^ in the absence of calcium and 383 ± 93 nm s^−1^ in the presence of 100 μM Ca^2+^and 2 μM CaM. The same behavior is observed in the filament gliding assay where the movement of actin filaments (413 ± 128 nm s^−1^) stalled in the presence of 100 µM Ca^2+^. Excess CaM partially restored the gliding velocity to 264 ± 92 nm s^−1^ (Fig. 6c). In the optical trap, no actomyosin interaction could be observed in the presence of Ca^2+^, indicating that the actin–myosin interaction is greatly reduced under pN-range loads. Excess CaM restored the actin–myosin interaction; however, most interactions were non-processive. If processive interactions were observed, the velocity and run length vs force dependence of the processive myosin-5B molecule was indistinguishable from the behavior in the absence of Ca^2+^ (Supplementary Fig. 5). In summary, these results indicate that Ca^2+^ completely abolishes the mechanical activity of myosin-5B. In the presence of Ca^2+^, a single myosin-5B molecule cannot processively move on actin either in unloaded conditions or in the presence of assistive or resistive forces. Excess CaM partially rescues processivity, thus suggesting a role of CaM in the calcium regulation of myosin-5B.

### Ca^2+^ does not affect ATPase but induces CaM dissociation

Based on the single-molecule observations, we hypothesized that Ca^2+^ has a direct effect on myosin motor activity. To test this hypothesis and to relate the single molecule observations to the ensemble kinetic measurements, we determined the enzymatic activity of myosin-5B under steady-state conditions. Actin activates the myosin-5B ATPase activity from a *k*_basal_ of 0.096 ± 0.0069 s^−1^ to a *k*_cat_ of 6.96 ± 0.14 s^−1^. The *K*_app_ is 1.34 ± 0.16 µM at 2000 µM ATP (Fig. 6d).

To relate ensemble solution measurement and single-molecule motility assays, we measured the ATP-dependence of the steady-state ATPase activity of myosin-5B. Increasing substrate concentrations increase the ATPase activity of myosin-5B to a *k*_cat,ATP_ of 6.41 ± 0.14 s^−1^ with a *K*_app,ATP_ of 59.07 ± 6.95 µM at a constant F-actin concentration of 30 µM (Fig. 6e). The *K*_app,ATP_ is in good agreement to the respective parameter (22 ± 2 µM) determined from the ATP-dependence of the velocity obtained from single-molecule motility assays (Fig. 2a).

Next, we measured the effect of Ca^2+^ on the steady-state ATPase activity of myosin-5B. Ca^2+^ does not affect the steady-state ATPase activity of myosin-5B (that in our experiments lacks the tail domain) over several orders of magnitude (Fig. 6f). To address whether Ca^2+^ would cause dissociation of CaM from all or a subset of the six myosin-5B IQ motifs and therefore uncouple the enzymatic and mechanical activity of myosin-5B, we performed an F-actin cosedimentation assays. The results demonstrate that increasing Ca^2+^ concentrations dissociate ~25% of CaM with a *K*_D_ < 10 µM (Fig. 6g, Supplementary Fig. 6). The titrated *K*_D_ is similar to the one obtained from in vitro gliding assays data on myosin-5A^52^. Of note, this study did not attempt to identify which of the six CaMs bound to myosin-5B dissociates in the presence of excess Ca^2+^.

## Discussion

The use of single-molecule imaging and high-speed force-clamp optical tweezers enabled us to identify the molecular details underlying the mechanosensitivity of the molecular motor myosin-5B and its regulation by calcium in real time. This elementary information provides insights into the dynamic regulation of the motor in physiologically important processes including cargo transport in cells.

**Fig. 7:**
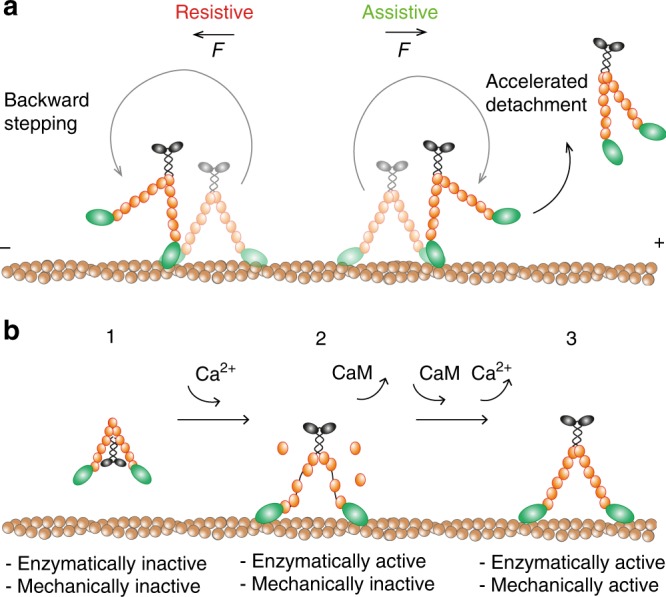
Proposed model of myosin-5B regulation by force and Ca^2+^. **a** In the presence of assistive forces, myosin-5B moves in the direction of the force at full speed by consecutive steps towards the plus actin end. However, assistive forces drastically reduce myosin run length as a consequence of accelerated detachment of the motor from actin. On the other hand, resistive forces slow down the motor and reduce its run length as a consequence of increased back stepping. **b** (1) In the absence of Ca^2+^, myosin-5B folds in an enzymatically and mechanically inactive conformation where head-tail interaction hinders binding to actin. (2) As Ca^2+^ concentration increases, myosin-5B switches to an open conformation that can bind to actin, thus becoming enzymatically active. However, Ca^2+^-induced detachment of 25% of CaM molecules results in loss of myosin processivity. (3) In the presence of excess CaM (2–5 μM), new CaM molecules are available for occupying the vacant binding sites on the light chains, thus restoring myosin capability to move along actin

We demonstrate with in vitro reconstituted assays that myosin-5B moves for several hundred nanometers under unloaded conditions on individual actin filaments. Myosin-5B has a large step size of 36 nm, approximating the actin pseudo-repeat. Together, these mechanical properties qualify myosin-5B as processive motor in the absence of load and agree with its transient kinetic properties under unloaded conditions^32^. However, myosin motors constantly experience forces and heterogeneity in the actin cytoskeleton^1^ that may influence their ability to move cargos as a single molecule. Our optical trapping measurements over a regime of 0.5–3 pN resistive and assistive forces show how myosin-5B might respond to loads in the crowded cell environment. Assistive loads result in moderate acceleration of the motor but in significant reduction of its run length (Fig. 4a, b). The reduced run length is mainly caused by disengagement of the motor from the actin filament, as indicated by the equal load sensitivity of both run length and detachment rate (*d*_*L*_^−^ ~ *d*_*k*_^−^) (Fig. 4c). On the other hand, resistive loads decrease both velocity and run length, nullifying both parameters at stall force ~2 pN and induce multiple backward stepping events at super-stall forces (Fig. 4a, b and Supplementary Fig. 3). Myosin run length decreases with resistive forces more rapidly than what would be expected from actin detachment only (*d*_*L*_^+^ > *d*_*k*_^+^), indicating that another mechanism must be responsible for the observed behavior. Interestingly, and opposed to myosin-5A, myosin-5B stepping rate accelerates under resistive loads, demonstrating that the reduced velocity and run length cannot be a consequence of slower stepping. Indeed, the ratio between the number of forward and backward steps (*N*_fwd_/*N*_bwd_) rapidly decreases with increasing force and is equal to one at the stall force, accounting for nullification of both velocity and run length. Therefore, load-dependence of the backward step conformational change seems to be the basis of the overall velocity and run length dependence on resistive force. Figure 7a shows a scheme that resumes the main features of myosin-5B mechanosensitivity.

A comparison of myosin-5B mechanosensitivity with that of its paralog myosin-5A shows similarities in the asymmetric force-velocity curve. Both myosin-5B and -5A velocities are fairly constant for assistive force, decrease monotonically in the 0–2.5 pN range of resistive force, and are reverted for resistive forces above 3 pN^48,50,53^. Such similarity is probably a consequence of a similar unsensitive stepping rate with assistive forces (Fig. 5e and ref. ^48^.) and increased backward stepping under resistive forces (Fig. 5d and refs. 47,53,). However, the strong and asymmetric reduction of myosin-5B run length with force sets an important difference with respect to myosin-5A, whose run length is unaffected by load in the −5 to 1.5 pN range^48^. Moreover, since myosin-5A run length is constant while its velocity decreases with resistive forces, the time that the protein spends bound to actin must increase with load, contrary to myosin-5B (Fig. 4c). Surprisingly, the mechanosensitivity observed here for myosin-5B is strikingly similar to what has been recently reported for the microtubule-based motor kinesin-1^54,55^. The qualitative features of load dependence of kinesin-1 for run length, actin detachment rate, velocity, and *N*_fwd_/*N*_bwd_ are very similar to myosin-5B^56^. Surprisingly, the accelerated forward stepping rate of myosin-5B under resistive force differes from both myosin-5A^48,50,51^ and kinesin-1^57^, indicating that a different gating mechanism must be at the base of myosin-5B stepping. Further experiments and detailed kinetic models will be needed to address this issue.

Myosin-5B mechanosensitivity is adapted to its proposed role as a transporter^34^ and regulator of the actin cytoskeleton^36,58^. The range of forces probed in our experiments are frequently experienced in living cells where other myosin motors apply assistive and resistive forces of similar magnitude^59^. Myosin-5B has been shown to drive the collective long-range transport of vesicles connected through an actin network towards the plasma membrane^36^. Such collective motion requires molecular mechanisms adapted for working in large ensembles. The strong reduction in run length and the accelerated actin detachment in the presence of assistive force might be one of the mechanisms to limit energy consumption when the vesicle cargo is moved towards the proper direction by either (i) other (myosin) motors connected to the same cargo vesicle or (ii) global movements of the cargo-actin network^36^. Conversely, resistive forces imposed for example by the cargo in the dense cellular actin cytoskeleton or by motors with opposite directionality^60^, slow down myosin-5B movement and decrease its run length, result in backward stepping and eventually the detachment of myosin from the actin filament. Although backward stepping is favored by increasing forces compared to actin detachment (*d*_fwd/bwd_^+^ > *d*_*k*_^+^), the time that myosin-5B spends bound to F-actin decreases with resistive forces. This feature further suggests that myosin-5B is not a strong transporter as a single motor compared to myosin-5A. The large difference in the mechanosensitivity of myosin-5B run length compared to -5A is predicted to affect the collective motion of multiple myosin-5B motors^45^. Multiple Kinesin-1 motors have been shown to produce significantly longer runs than a single motor^61–64^, although the system effectively behaves as though a single-motor attachment state dominates motility^65,66^. Likely, the enhanced disengagement of kinesin-1 under assistive loads favors the binding of just one motor at the time, thus reducing ATP consumption but providing a second motor if the first one detaches to increase run lengths^54^. Owing to the similar mechanosensitivity of myosin-5B run length, a similar mechanism might be at the basis of its collective behavior. However, coupling between multiple motors is a complex issue in which multiple factors play important roles^67^ and futher experiments would be needed to asses this point.

Lastly, our detailed biophysical characterization led to a refined mechanistic model of myosin-5B processivity and activation during the Ca^2+^-dependent trafficking of recycling endosomes in the dense actin cytoskeleton (Fig. 7b). Ca^2+^ has been previously shown to relieve the autoinhibition of the actin activated ATPase of myosin-5B in vitro and enhance the interaction of myosin 5B with binding partners in in vivo co-immunoprecipitation experiments by disrupting the head:tail interaction^34^. However, this study did not explore the effect of Ca^2+^ on the ability of myosin 5B to move actin filaments.

Here we find that micromolar Ca^2+^ levels induce the dissociation of ∼25% of the CaM molecules bound to myosin-5B and the Ca^2+^-induced CaM dissociation prevents the motor from moving processively along single actin filaments (Fig. 6). Therefore, physiological transient changes in free Ca^2+^ relieve myosin-5B head–tail interaction, allowing myosin-5B to engage with a cargo, but (i) result in the dissociation of a subset of the CaMs and (ii) inhibits myosin-5B movement on actin. Only after free Ca^2+^ dissipation or diffusion/transport of the myosin-5B:cargo complex in regions with low free Ca^2+^, myosin-5B can bind to actin and transport the cargo to its final destination. The Ca^2+^ regulation mechanism that we observed for myosin-5B is similar to what has been previously reported for myosin-5A^52,68,69^ and partially supports a model proposed for myosin-5B trafficking of recycling endosomes and AMPA receptors in dendritic spines^34^. In that model, due to the steep gradient of Ca^2+^ concentration in the spine that decreases towards the spine base, only myosin-5B motors that are at the base of the spine can actively move out to the distal membrane. However, it remains elusive how myosin-5B might deliver a cargo to the distal membrane, where Ca^2+^ levels are high. Here we found that the presence of CaM in the cytosol could potentially rescue myosin-5B motility and allow it to navigate also in regions with high Ca^2+^ levels. However, this would unlikely happen in the cell because of the reduction of available CaM with increasing Ca^2+^^70^. The measured levels of free CaM in the presence of micromolar levels of Ca^2+^ are far below the *K*_D_ for CaM binding to myosin-5B which we have measured. Therefore, our results suggest that myosin-5B can actively translocate cargoes in living cells only during time intervals following dissipation of Ca^2+^ spikes.

## Methods

### DNA constructs and proteins production and purification

A cDNA construct encoding for amino acids 1–1095 of murine myosin-5B heavy meromyosin (Accession number NM_201600.2, MW 127 kDa) or myosin-5B heavy meromyosin with a C-terminal Avi-tag was inserted in a modified pFastBac1 vector encoding a C-terminal Flag-tag with standard cloning techniques (Supplementary Table 1). Untagged rat CaM cDNA (Accession number NP_114175.1; 17 kDa) that is at the protein level 100% identical with murine CaM was expressed from pFastBac1. Recombinant baculovirus generation and gene expression were performed as recommended by the manufacturer (Thermo Fisher Scientific). For protein production, *Sf*9 insect cells from ThermoScientific (B82501) were infected with recombinant baculoviruses encoding myosin-5B and CaM. Myosin-5B heavy meromyosin was purified via Flag capture^71^ followed by size exclusion chromatography on a HiLoad Superdex 16/600 pg (GE Healthcare Life Sciences). Avi-tag myosin-5B heavy meromyosin was purified via Flag capture^71^ followed by ion exchange chromatography through Q-Sepharose Fast Flow resin (GE Healthcare Life Sciences). For single molecule motility assays, myosin-5B with a C-terminal Avi-tag was biotinylated on the Flag-resin through incubation with BirA biotin ligase and d-biotin (Avidity). Biotinylated myosin-5B was then conjugated to a steptavidin-functionalized quantum dot (QD, 655 nm emission wavelength, Q10123M Molecular Probes) at a 1:5 molar ratio to ensure that a Qdot was conjugated to a single motor^72^, by incubation in buffer containing 10 mM MOPS pH 7.3, 0.5 M NaCl, 0.1 mM EGTA, 3 mM NaN_3_, 4.4 µM alpha-casein, 2 µM CaM, and 6 mM DTT for >30 min on ice. Free streptavidin sites were saturated during a 10 minutes incubation with 1 mM D-biotin prior to the assay. G-actin for cosedimentation, ensemble kinetic, and gliding assays was prepared from rabbit skeletal muscle acetone powder (Pel-Freez Biologicals)^73^, while G-actin for single molecule motility assay and ultrafast force-clamp spectroscopy was bought from Cytoskeleton (AKL99). F-actin for single molecule experiments was polymerized and labeled overnight with 10 μM rhodamine phalloidin in 1× actin polymerization buffer (Cytoskeleton, BSA02) and 20 mM dl-dithiothreitol.

### Single molecule in vitro motility assay

All components were loaded into a flow chamber of about 20 µL volume coated with 1% w/v nitrocellulose/amyl acetate on the coverslip surface. In total, 2 mg ml^−1^*N*-ethyl-maleimide inactivated myosin-2 (NEM-M2)^74^ was loaded into the flow chamber and incubated for 1 min. Then 23.3 nM rhodamine phalloidin F-Actin in motility buffer (MB: 20 mM MOPS pH 7.4, 1 mM MgCl_2_, 0.1 mM EGTA, 50 mM KCl, and 1 mM DTT) was introduced. After careful washing with MB, 0.1 nM QD-myosin-5B in MB supplemented with 1.2 µM glucose oxidase, 0.2 µM catalase, 4.2 µM alpha-casein, 17 mM glucose, and 20 mM DTT was introduced. Experiments were performed in the aforementioned buffer supplemented with 2 µM CaM (unless otherwise specified) and 0.3–1000 µM ATP.

### Single-molecule motility data acquisition

All motility experiments were performed on an inverted fluorescence microscope (Nikon ECLIPSE TE300) equipped with a 532 nm laser (Coherent Sapphire) for rhodamine excitation (~3 mW on the sample) and a 488 nm Laser Physics argon laser for QDs-655 nm excitation (~3 mW on the sample). Images were acquired in total internal reflection configuration, through Nikon Plan Apo TIRF, 1.45 oil immersion objective. 91 nm pixel size images were obtained by projecting the fluorescence signal onto a iXon 3 EMCCD camera, after an additional 3× magnification through an achromatic doublet telescope. At the beginning of each record, one F-actin image was acquired and used afterwards to overlay the trajectory of the moving QD with its correspondent actin filament (Fig. 1b). Depending on the ATP concentration, different integration times (50–100 ms) were used at constant EM gain = 300.

### Single-molecule motility data analysis

High precision localization of single QDs was performed by a custom-made Matlab algorithm that automatically detects the fluorescent emitter within a region of interest (ROI) and fits a bidimensional Gaussian function to its intensity profile^41^. Localization accuracy in single molecule motility experiments was around 4 nm (100 ms integration time, 300 EM gain, 3 mW laser power on the sample), calculated as standard deviation of immobile QDs on actin filaments over 20 frames. Myosin trajectories were derived from *x*,*y* coordinates of QDs moving along actin filaments for more than ten frames. The run length was calculated from *x–y* trajectories. The actual run length was calculated by interpolating the trajectories to improve run length accuracy. The velocity was calculated as the run length divided by the run duration. Velocity and run length reported in Fig. 2a, b were obtained from 127 QDs (molecules) out of 135 moving QDs (8 QDs moved for <10 frames and were excluded from the analysis). Data in Fig. 6a, b were obtained from 12 molecules. The lower limit on run length measurement was determined by the product between myosin velocity and integration time, varying between 16 nm at 5 μM ATP and 55 nm at 100 μM ATP. The step size was measured from data sets at 0.3 µM ATP. Trajectories were visually inspected to isolate stepping events and the step size was calculated as the distance between the average position before and after the step. The reported value was averaged over 42 steps from three molecules. The number of processive runs per second reported in Fig. 6b was calculated as the number of QDs moving along actin for >10 frames divided by the total acquisition time. The acquisition time included several fields of view within a sample to average variability in different regions of the microscope slide.

### Ultrafast force-clamp spectroscopy setup

The experimental apparatus and ultra-fast force-clamp spectroscopy are described in detail elsewhere^38,40^. Briefly, the experimental setup comprises an inverted optical microscope combined with double optical tweezers and fluorescence microscopy with single molecule sensitivity. The system is stabilized to <1 nm with both passive and active stabilization^75,76^. The pedestal bead is used as a reference for the sample coordinates correction against thermal drifts and low-frequency noise obtained by moving piezo translators. The two traps can be moved along *x* by acousto-optic deflectors and the position of the trapped beads is measured using quadrant detector photodiodes placed in a plane conjugate to the back focal plane of the condenser^77^. The force *F* applied on each bead is measured from the displacement of the bead from the trap center (*x*) and from a calibration of the trap stiffness (*k*), as *F* = −*kx*. Before each experiment, *k* was calibrated over the entire range of trap positions used during the experiment, with a power spectrum method^78^. Trap stiffness in the range of 0.03–0.14 pN nm^−1^ were used in the experiments. A custom software written in Labview controlled the force-feedback system and data acquisition through an FPGA board (NI-PCI-7830R), running at 200-kHz sample rate.

### Ultrafast force-clamp spectroscopy sample preparation

All components for trapping experiments were loaded into a flow chamber coated with 1.2 µm silica beads in 0.01% v/w nitrocellulose/amyl acetate on the coverslip surface^40^. An aliquot of 1 mg ml^−1^ biotinylated BSA was first incubated for 5 min, followed by 1 mg ml^−1^ streptavidin for 5 min. An aliquot of concentration of 3 nM biotinylated myosin-5B in 10 mM MOPS pH 7.3, 0.5 M NaCl, 0.1 mM EGTA, 3 mM NaN_3_, 4.4 µM alpha-casein, 2 µM CaM, and 6 mM DTT was incubated for 5 min on the surface. The flow cell was washed with 1 mg ml^−1^ biotinylated BSA supplemented with 2 µM CaM. Final mix for experiments was composed with 0.005% alpha-actinin functionalized beads 1 µm diameter^79^, 1 nM rhodamine phalloidin F-Actin in Imaging Buffer (IB: 25 mM MOPS pH 7.2, 25 mM KCl, 4 mM MgCl_2_, 1 mM EGTA, 1.2 µM Glucose-Oxidase, 0.2 µM catalase, 17 mM glucose, and 20 mM DTT) supplemented with 2 µM CaM (unless otherwise specified) and 100 µM or 1 mM ATP. Measurements were carried out after sealing the flow chamber with high vacuum grease. Under these conditions, approximately 1 in 3 silica beads interacted with the actin filament, providing evidence that the large majority of the beads contained at most one myosin-5B molecule. Experiments shown in Figs. 4, 5 were carried out at 100 µM ATP, where myosin-5B moves close to its saturating velocity (~0.8*v*_max_, see Fig. 2a), but provided better signal-to-noise ratio for step detection than measurements performed at 1 mM ATP. Experiments performed at 1 mM ATP did not show significant differences in the force-dependent behavior of myosin-5B (Supplementary Fig. 7). Data were recorded for five molecules at 100 µM ATP and eight molecules at 1 mM ATP.

### Ultrafast force-clamp spectroscopy data analysis

Analysis of ultra-fast force-clamp data for non-processive motors is extensively described elsewhere^38,40^. Briefly, detection of interactions was based on the variation of the dumbbell velocity using a threshold-based method that ensured false events below 1%^38^. The analysis method was modified to allow the detection of steps within processive runs, and the measurement of the step size, run length, and velocity. The detection of steps in processive runs was based on the change in velocity, as caused by myosin stepping. Threshold crossing was allowed in both directions, to detect both forward and backward steps. Subsequent steps were assigned to belong to the same run when the time interval between steps was <3 ms and the amplitude of the step size <90 nm (to allow two steps occurring within the time resolution of the step detection method to be detected as one). Steps out of these limits were assigned to another run.

### Run length correction for assistive forces

In ultrafast force-clamp spectroscopy, when the dumbbell is suspended in solution, it alternatively moves in opposite directions according to the direction of the applied force. The force direction is reversed when the trailing bead reaches the edge of the oscillation range (*D*) set by the operator. With reference to Supplementary Fig. 8, when the force is directed towards positive displacement, the feedback waits for the bead to reach the upper edge of the oscillation range to switch the force in the negative direction, and vice versa. When myosin binds and moves the filament in one direction (positive in Supplementary Fig. 8), it can happen that it moves the bead up to the (upper) edge of the oscillation range. In such a case, if the force was directed towards positive displacement (assistive, defined negative here following the common usage), the dumbbell reaches the upper oscillation edge where the force is reversed, so that the myosin run under assistive force is interrupted (arrows in Supplementary Fig. 8). Therefore, in case of assistive forces, the run length is limited by the amplitude of the dumbbell oscillation *D*. On the other hand, if the force was directed towards negative displacement (resistive), the processive stepping of the protein prevents the dumbbell from reaching the force inverting position (on the lower edge of the oscillation, in this case). Therefore, the length of the processive run is not limited by the oscillation range for forces resisting myosin movement (Supplementary Fig. 8, resistive).

Therefore, run lengths under assistive force were corrected by calculating the real run length value *L* from the expected (measured) average run length value < *L*_*m*_ > under our experimental constrains. We assume that the real myosin-5B run length is exponentially distributed (as confirmed by its distribution under resistive force, which is not constrained, see Supplementary Fig. 9), so that its survival function (i.e., the probability of having a run longer than *x*) is given by

1 $$S_{{L}} = {{\mathrm e}}^{ - \frac{x}{{{L}}}}$$

If we also assume that the probability of myosin binding to actin is constant along the dumbbell oscillation between 0 and *D*, and zero elsewhere, the associated survival function is given by

2 $$S_x = \left\{ {\begin{array}{*{20}{c}} {1 - \frac{x}{D},x \le D} \\ {0,x \, > \, D} \end{array}} \right.$$

Therefore, the probability that myosin generates a run longer than *x* is the product of the two probabilities (Eq. (1)) and (Eq. (2))

3 $$S_{L} \cdot S_x = \left\{ {\begin{array}{*{20}{c}} {{\mathrm{e}}^{ - \frac{x}{{L}}}\left( {1 - \frac{x}{D}} \right),x \le D} \\ {0,x \, > \, D} \end{array}} \right.$$

Form Equation (3) the probability density function can be calculated

4 $$p\left( x \right) = \left\{ {\begin{array}{*{20}{c}} {{\mathrm{e}}^{ - \frac{x}{{L}}}\left( {\frac{1}{{L}} + \frac{1}{D} - \frac{x}{{D \cdot L}}} \right),x \le D} \\ {0,x \, > \, D} \end{array}} \right.$$

From Equation (4), the expected run length value < *L*_*m*_ > can be calculated as

5 $$< L_m > = \mathop {\int }\limits_{ - \infty }^{ + \infty } x \cdot p\left( x \right){\mathrm d}x = \frac{1}{D}\left[ {L^2\left( {{\mathrm{e}}^{ - \frac{D}{{L}}} - 1} \right) + L \cdot D} \right]$$

From the measured run length < *L*_*m*_ > we calculated the real run length *L* by numerically solving Eq. (5).

### Cosedimentation assay

In total, 3 µM myosin-5B heavy meromyosin was incubated with 15 µM F-actin in buffer containing 10 mM MOPS pH 7.2, 150 mM NaCl, 0.1 mM EGTA, 3 mM NaN_3_, and CaCl_2_ to final free concentrations of ~0 µM, 10 µM, 50 µM, 100 µM, 150 µM, 200 µM, and 400 µM. The samples were incubated at room temperature for 15 min and spun at 100,000 × *g* for 20 min at a temperature of 4 °C in a TLA-100 rotor in a Beckman MAX-XP ultracentrifuge. The supernatant was removed and the pellet resuspended in the original volume of assay buffer. NuPAGE LDS Sample Buffer (Thermo Fisher) was added to the samples and supernatant and pellet fractions resolved on a 4–12% Bis–Tris gel (Thermo Fisher) with MES running buffer at 200 V for 35 min, stained with PageBlue (Thermo Fisher), and destained in water. The gel was scanned on an Odyssey scanner (Li-Cor Biosciences) and analyzed by densitometry with Fiji^80^.

### Ensemble kinetic and in vitro gliding assay

The actin-activated steady-state ATPase activity was measured with the NADH-coupled assay as described in buffer containing 10 mM MOPS pH 7.0, 50 mM NaCl, 2 mM MgCl_2_, 2000 µM ATP, 0.15 mM EGTA, 40 U ml^−1^l-lactic dehydrogenase, 200 U ml^−1^ pyruvate kinase, 200 μM NADH, 1000 µM phoshoenolpyruvate, and 50–80 nM myosin-5B in a spectrophotometer (Cary 60, Agilent Technologies) at 25 °C^71^. To test the effect of ATP on the steady-state ATPase activity, the substrate concentration was increased from 0 to 2 mM at a fixed F-actin concentration of 30 µM. Similarly, the free Ca^2+^ concentration was increased from pCa 3 to 9 at a fixed F-actin concentration of 30 µM and an ATP concentration of 2000 µM. The in vitro gliding assay was performed in buffer containing 20 mM MOPS pH 7.4, 50 mM KCl, 5 mM MgCl_2_, 0.1 mM EGTA, 1 mM ATP, 25 µg ml^−1^ glucose oxidase, 45 µg ml^−1^ catalase, 2.5 mM glucose, and 50 mM DTT at a temperature of 30 °C^71^. The gliding speed of actin filaments was analyzed with the CellTrak (Motion Analysis)^42^ excluding filaments that showed a velocity standard deviation/velocity ratio larger than 0.33.

### Data availability

Data supporting the findings of this manuscript are available from the corresponding author upon reasonable request.

## Electronic supplementary material

Supplementary Information
