# Supplementary material for: Dissecting myosin-5B mechanosensitivity and calcium regulation at the single molecule level
Source: Nat Commun. 2018 Jul 20;9:2844. doi: 10.1038/s41467-018-05251-z (PMC6054644; doi:10.1038/s41467-018-05251-z)
Supplement: Supplementary file 1 — Supplementary Information [file 41467_2018_5251_MOESM1_ESM.pdf]

Dissecting myosin-5B mechanosensitivity and calcium regulation at the single molecule level

Gardini et al.

Supplementary Information

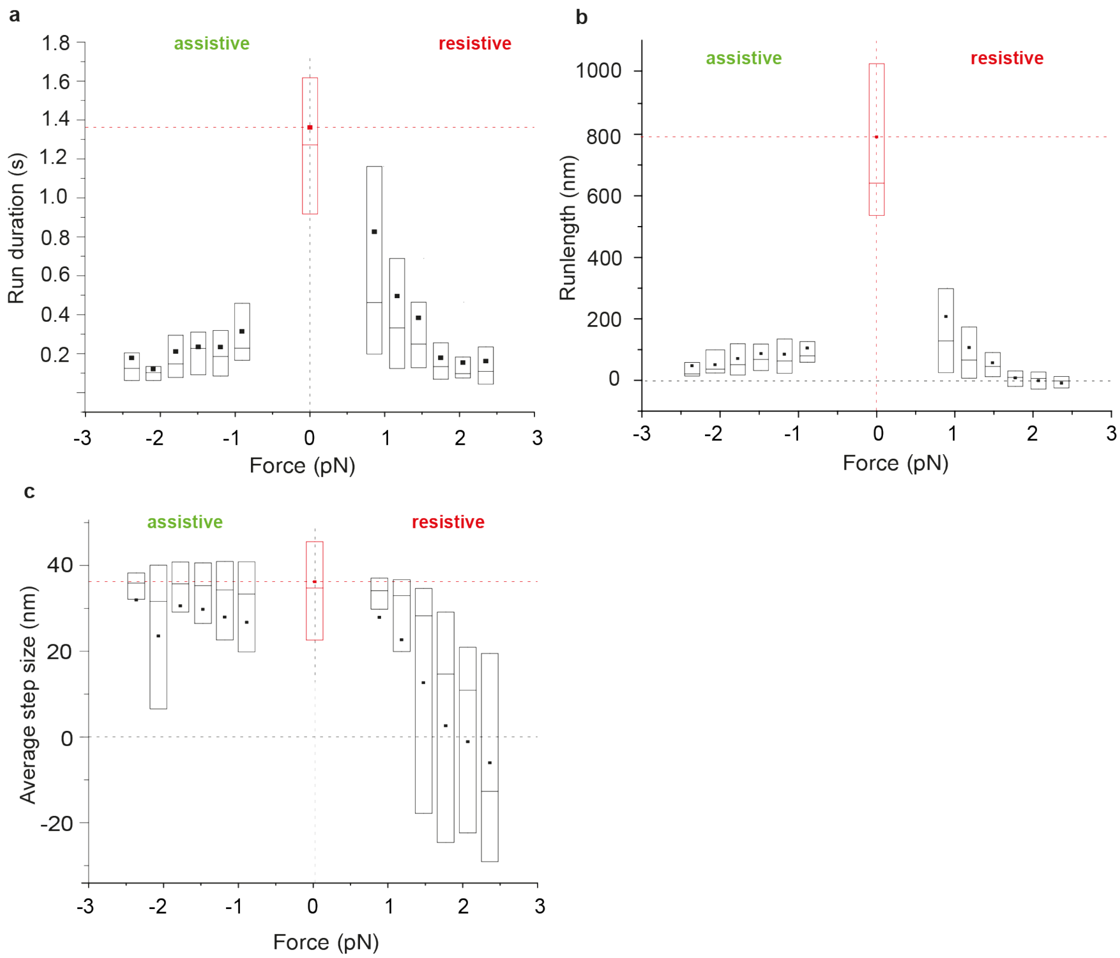

Supplementary Figure 1. Box plots of run duration (a) and run length (b) and step size (c) versus force. Box plots show the distribution of data underling the average values reported in Fig. 4c **(a)**, 4b **(b)** and 5a **(c)**, respectively. Detachment rates shown in Fig. 4c are obtained from the run duration shown in panel (a). Box edges correspond to quartiles (25% and 75% percentiles), the crossing line represents the median value, while the solid dot represents the average value.

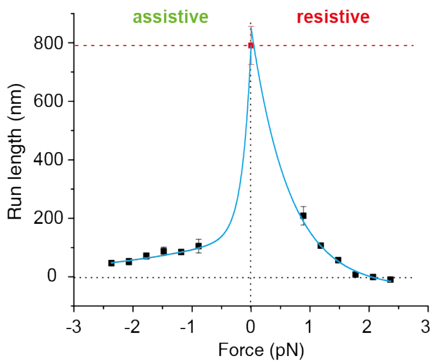

Supplementary Figure 2. Double-exponential fit of myosin-5B run length force-dependence. Force dependence of myosin-5B average run length at 100 μM [ATP]. Red square is the unloaded run length measured with the single molecule motility assay. Run lengths under assistive forces were fitted with a double-exponential model equation $L = L_0^1 \exp\left(-\frac{F \cdot d_L^1}{k_B \cdot T}\right) + L_0^2 \exp\left(-\frac{F \cdot d_L^2}{k_B \cdot T}\right)$. Fitted parameters are L_0^1 = 644±53 nm, L_0^2 = 147±23 nm, d_L^1 = 27±66 nm, d_L^2 = 1.9±0.4 nm. Error bars, s.e.m., *n*=756. The low force-dependent transition (L_0^2 = 147±23 nm; d_L^2 = 1.9±0.4 nm) corresponds to the single-exponential fit reported in Fig. 4b (L_0^- = 148±12 nm, d_L^- = 1.9±0.3 nm). The large distance parameter of the highly force-dependent transition (d_L^1 = 27±66 nm) indicates that this transition is associated with large-scale rearrangements of the myosin molecule.

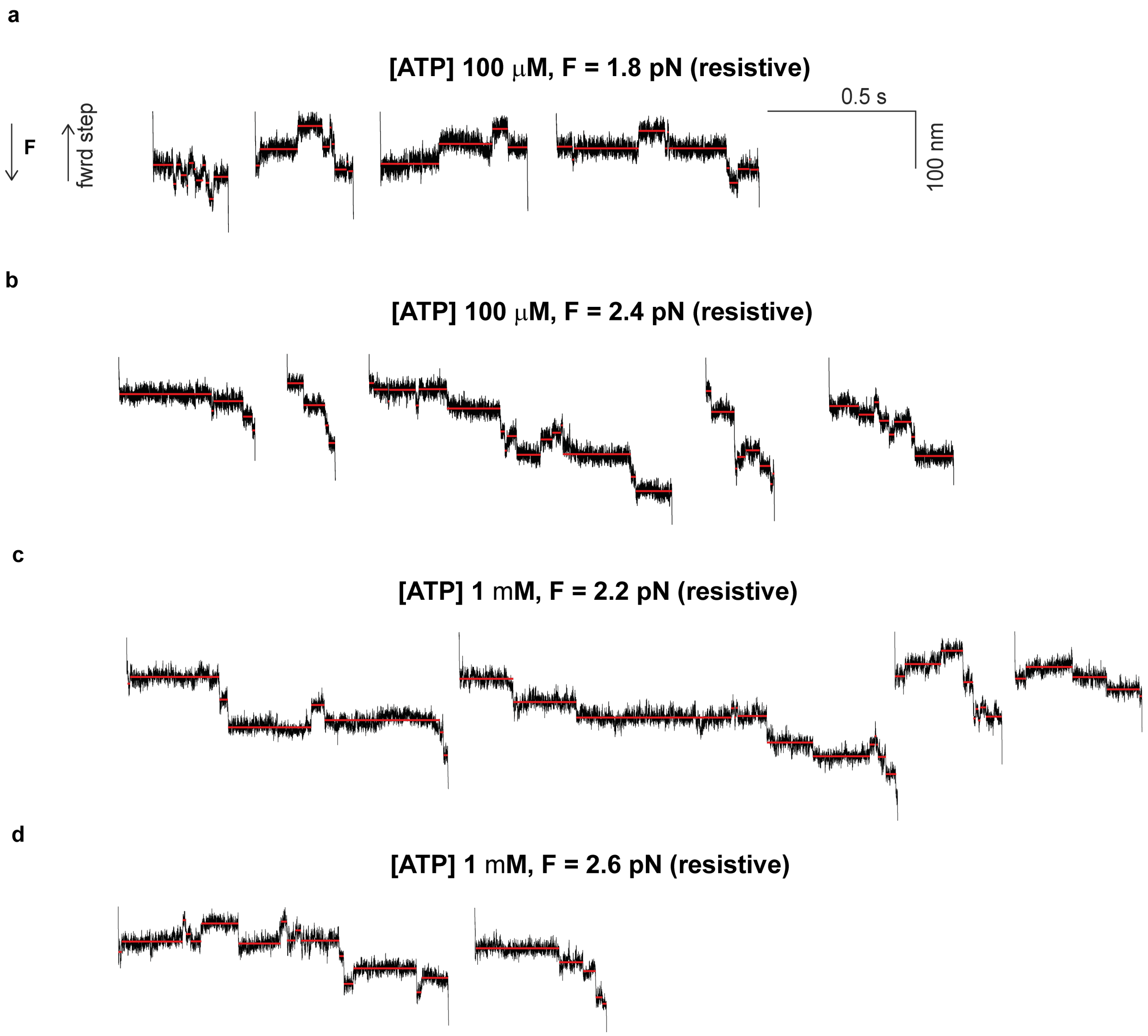

Supplementary Figure 3. Representative position records of myosin-5B stepping at stall and super-stall forces Representative position records showing myosin-5B runs at **(a)** stall force, 100 μM [ATP]; **(b)** super-stall force, 100 μM [ATP]; **(c-d)** super-stall forces at 1 mM [ATP]. Arrow ‘F’ indicates the direction of the applied force, while the direction of the forward step is indicated by the ‘fwd’ arrow. Consecutive multiple backward steps are visible at all super-stall forces.

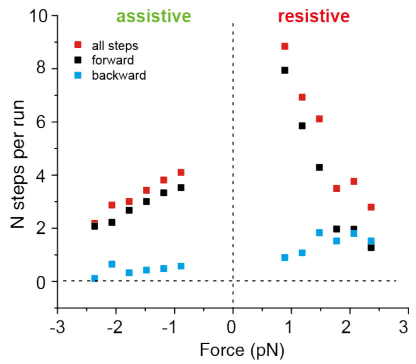

Supplementary Figure 4. Force-dependence of number of steps per run. The plot shows the number of forward (black squares) and backward (cyan squares) steps per run and the total number of steps per run (red squares) as a function of load. [ATP]=100 μM. The total number of steps per run for resistive forces is higher than for assistive forces for forces <1.75 pN. Above the stall force, the number of forward steps decrease significantly, but this is partially compensated by increase in backward stepping.

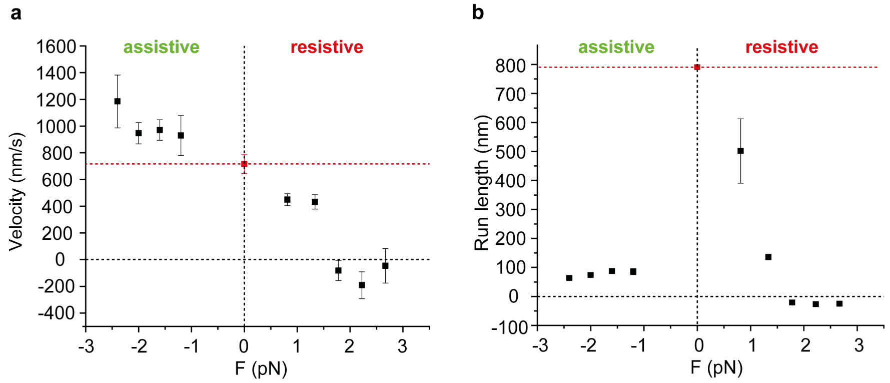

Supplementary Figure 5. Force-dependence of myosin-5B velocity and run length in the presence of Ca^{2+} and CaM. Force dependence of myosin-5B average velocity (**a**) and run length (**b**) in the presence of 100 μM Ca^{2+} and 2 μM CaM. Red squares are the unloaded velocity (a) and run length measured with the single molecule motility assay, $n_{unloaded}$ = 13. [ATP]= 1000 μM. Error bars, s.e.m. n = 864.

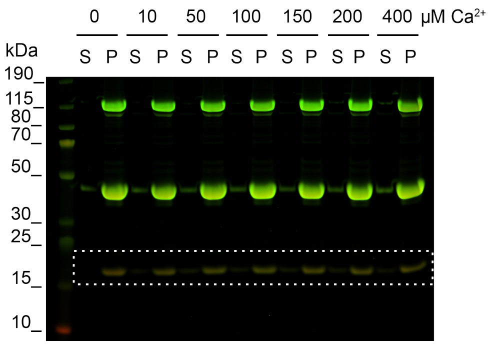

Supplementary Figure 6. Micromolar Ca^{2+} concentrations induce partial dissociation of CaM from myosin-5B light chain. F-actin cosedimentation assays show that increasing concentrations of Ca^{2+} partially dissociate CaM from myosin-5B. The boxed area highlights CaM. The abbreviations S and P refer to supernatant and pellet fraction.

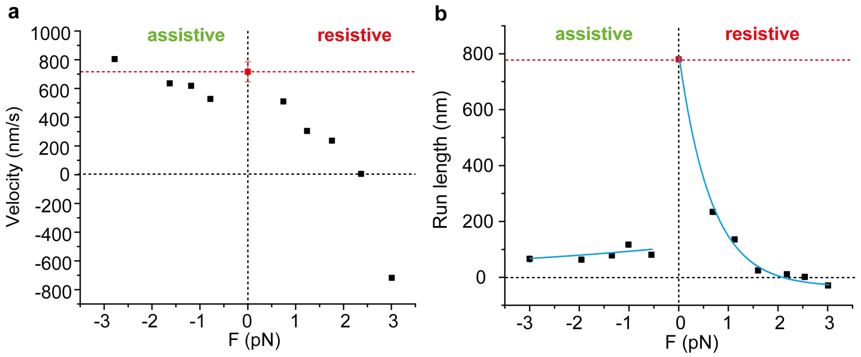

Supplementary Figure 7. Force-dependence of myosin-5B velocity and run length at 1 mM [ATP]. Force dependence of myosin-5B average velocity (**a**) and run length (**b**) at 1 mM [ATP]. Red squares are the unloaded velocity (a) and run length (b) measured with the single molecule motility assay, $n_{unloaded}$ (a,b) = 13. Cyan curves in (b) are fits of the exponential model equations $L = L_0^{\pm}\exp\left(-\frac{F\cdot d_L^{\pm}}{k_B\cdot T}\right)$ to data, where F is the absolute value of the force, k_B the Boltzmann constant, T the temperature, and + or – superscripts indicate free parameters under positive or negative force, respectively. Fitted parameters are L_0^- = 106±21 nm, L_0^+ = 815±15 nm, d_L^- = 0.7±0.4 nm, d_L^+ = 6.2±0.3 nm. n = 2375. Error bars, s.e.m.

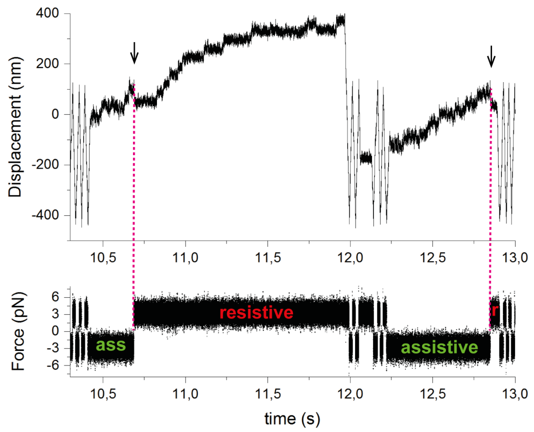

Supplementary Figure 8. Force inversion during myosin runs. When myosin binds and moves the filament in the positive direction under assistive force , it can happen that it reaches the edge of the oscillation range where the force is reversed (indicated by the arrows), so that the myosin run under assistive force is interrupted. Contrary, under resistive force, myosin processive stepping prevents the dumbbell from reaching the force inversion point. Therefore, run lengths are not limited by the oscillation range for resistive forces.

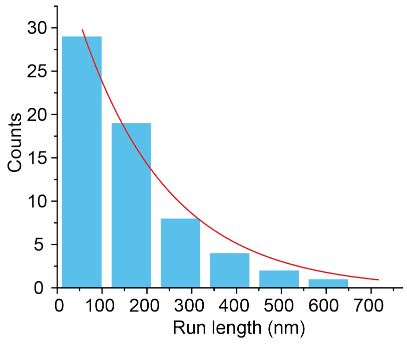

Supplementary Figure 9. Myosin-5B run length under resistive force is exponentially distributed. Histogram of myosin-5B run lengths under 1.2 pN resistive force and 100 µM [ATP]. Red curve is the exponential fit to the data.

Supplementary Table 1. Primers and oligonucleotides.

**Primer name**	**Sequence**	**Description**
pmMVb-F2-SpeI	cgactagtatgtcgtacagcgagctctacacccgg	Primer Myosin Vb – Forward – SpeI restriction site
pmMVb-HMM-R-NotI	atgcggccgcctggtcggcattcatgatctgggtcct	2 cc rev. primer + NotI – 3784-3807 CDS seq., 1262-1269 protein seq.
**Oligo name**	**Sequence**	**Description**
Bio-F-NotI	ggccgcaggtggcggtctgaacgacatcttcgaggctcagaaaatcgaatggcacgaagc	Bio-F-NotI and Bio-R-NotI were annichilated and then ligated at N-terminus to MVb digested with NotI
Bio-R-NotI	ggccgcttcgtgccattcgattttctgagcctcgaagatgtcgttcagaccgccacctgc	
